# MicroRNA profiling implicates the insulin-like growth factor pathway in bleomycin-induced pulmonary fibrosis in mice

**DOI:** 10.1186/1755-1536-6-16

**Published:** 2013-08-29

**Authors:** Lisa Honeyman, Mark Bazett, Tomasz G Tomko, Christina K Haston

**Affiliations:** 1 Meakins-Christie Laboratories and the Department of Human Genetics, McGill University, Montreal, QC, Canada; 2 Meakins-Christie Laboratories, Department of Medicine, McGill University, 3626 St. Urbain, Montreal, QC H2X 2P2, Canada

**Keywords:** Pulmonary fibrosis, microRNA, Bleomycin, Insulin-like growth factor, Pathway analysis, Mouse model

## Abstract

**Background:**

Idiopathic pulmonary fibrosis is a disease characterized by alveolar epithelial cell injury, inflammatory cell infiltration and deposition of extracellular matrix in lung tissue. As mouse models of bleomycin-induced pulmonary fibrosis display many of the same phenotypes observed in patients with idiopathic pulmonary fibrosis, they have been used to study various aspects of the disease, including altered expression of microRNAs.

**Results:**

In this work, microRNA expression profiling of the lungs from treated C57BL/6J mice, relative to that of untreated controls, was undertaken to determine which alterations in microRNAs could in part regulate the fibrosis phenotype induced by bleomycin delivered through mini-osmotic pumps. We identified 11 microRNAs, including miR-21 and miR-34a, to be significantly differentially expressed (*P* < 0.01) in lungs of bleomycin treated mice and confirmed these data with real time PCR measurements. *In situ* hybridization of both miR-21 and miR-34a indicated that they were expressed in alveolar macrophages. Using a previously reported gene expression profile, we identified 195 genes to be both predicted targets of the 11 microRNAs and of altered expression in bleomycin-induced lung disease of C57BL/6J mice. Pathway analysis with these 195 genes indicated that altered microRNA expression may be associated with hepatocyte growth factor signaling, cholecystokinin/gastrin-mediated signaling, and insulin-like growth factor (IGF-1) signaling, among others, in fibrotic lung disease. The relevance of the IGF-1 pathway in this model was then demonstrated by showing lung tissue of bleomycin treated C57BL/6J mice had increased expression of *Igf1* and that increased numbers of Igf-1 positive cells, predominantly in macrophages, were detected in the lungs.

**Conclusions:**

We conclude that altered microRNA expression in macrophages is a feature which putatively influences the insulin-like growth factor signaling component of bleomycin-induced pulmonary fibrosis.

## Background

Idiopathic pulmonary fibrosis (IPF) is a progressive disease of the lung interstitium characterized by deposition of extracellular matrix, inflammatory cell infiltration, and fibroblast recruitment and hyperplasia which leads to impaired lung function and ultimately, respiratory failure [1,2]. While the etiology of IPF is unknown, many of the characteristics of this disease are mimicked by the mouse models of bleomycin-induced pulmonary fibrosis [2,3]. Mice treated with bleomycin display subpleural scarring characterized by cellular inflammatory cell infiltration and extracellular matrix deposition in the alveoli, as has been described in clinical cases of idiopathic pulmonary fibrosis [2,3]. Studies have shown bleomycin-induced pulmonary fibrosis is influenced by, among others, secretion of a variety of chemokines [4,5], recruitment of inflammatory cells [6], involvement of transforming growth factor β1 (TGF- β1) [7,8] and epithelial-mesenchymal transition [9].

Many of the subphenotypes involved in bleomycin-induced pulmonary fibrosis have been shown to be independently influenced by microRNAs, including inflammation [10], tissue repair [11,12], cell differentiation [13,14] and cell proliferation [15]. MicroRNAs are small non-coding RNA molecules of approximately 22 nucleotides that regulate gene expression through complimentary binding, usually to the 3′-untranslated region of target mRNAs. MicroRNAs reduce gene expression by causing disruption of mRNA stability or translation [16-18] and can significantly change cellular processes through both repression of important targets and repression of multiple targets within the same pathway/process [19]. A large number of pathologies are known to be influenced by microRNAs. For respiratory diseases these include cancer [20], asthma [21], chronic obstructive pulmonary disease [22], cystic fibrosis [23] and idiopathic pulmonary fibrosis [24-26].

Others have investigated the involvement of microRNAs in the development of bleomycin-induced pulmonary fibrosis using intratracheal [27-29] and intraperitoneal [30] treatment delivery models. Among those microRNAs previously shown to be perturbed in fibrosis are microRNA-21 (miR-21) [29], miR-29 [30], miR-200 [31], miR-154 [24], miR-199a-5p [28] and let-7d [26]. These microRNAs have been shown to affect the epithelial-mesenchymal transition [25,31] and are expressed in fibroblasts [28] and myofibroblasts [29,30]. A mini-osmotic pump model of bleomycin-induced pulmonary fibrosis has been shown to more appropriately model the human disease [3,32] but the contributions of microRNA to the development of the pulmonary fibrosis phenotype have not been investigated using this model.

In this report, we use a mini-osmotic pump model of bleomycin-induced pulmonary fibrosis to investigate whether unique microRNAs contribute to the observed phenotypic changes. We initially measured the microRNA signature of the lung tissue with a microarray, and evaluated the cellular site of expression of specific microRNAs. Through bioinformatic analyses of genes potentially regulated by the differentially expressed microRNAs, coupled with a documented gene expression profile [33], we identified putative biological functions and pathways, including insulin-like growth factor signaling, through which these microRNAs may affect the bleomycin-induced lung response.

## Results

### Bleomycin-induced lung phenotype

**Figure 1:**
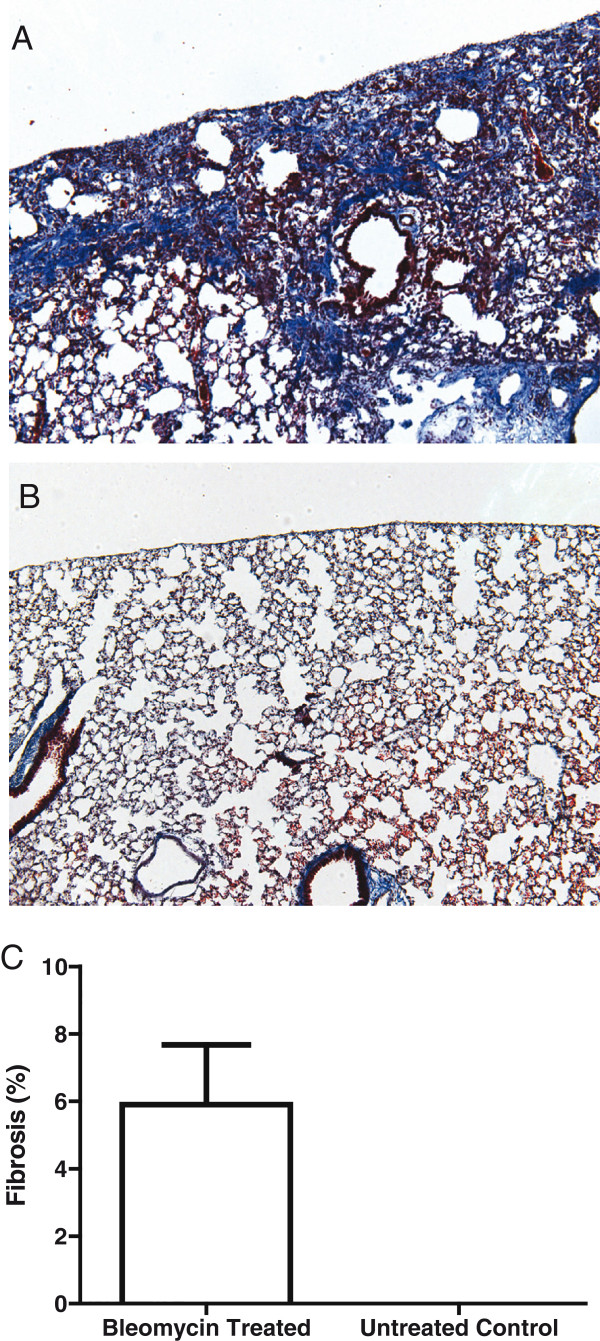
**Bleomycin-induced pulmonary fibrosis phenotype of C57BL/6J mice.** Mice were exposed to 100 U/kg of bleomycin through a mini-osmotic pump and euthanized six weeks later. Control mice were not treated. Images of left lung histological sections stained with Masson’s trichrome; magnification 100×. **(A)** C57BL/6J with regions of subpleural pulmonary fibrosis, as indicated by the blue collagen streaks. **(B)** C57BL/6J control. **(C)** Average fibrosis ± standard deviation of n = 3 mice per group.

Bleomycin treatment by mini-osmotic pump produced a pulmonary fibrosis in C57BL/6J mice consisting of regions of subpleural atelectasis, at six weeks post treatment, as shown in Figure 1A, which is consistent with previous reports of this model [3,33,34]. On average, the fibrotic scar covered 5.9 ± 1.8% of the lung in bleomycin treated C57BL/6J mice, while fibrosis was not evident in untreated control mice, as shown in Figure 1.

### MicroRNAs are differentially expressed in the bleomycin treated mouse lung

**Figure 2:**
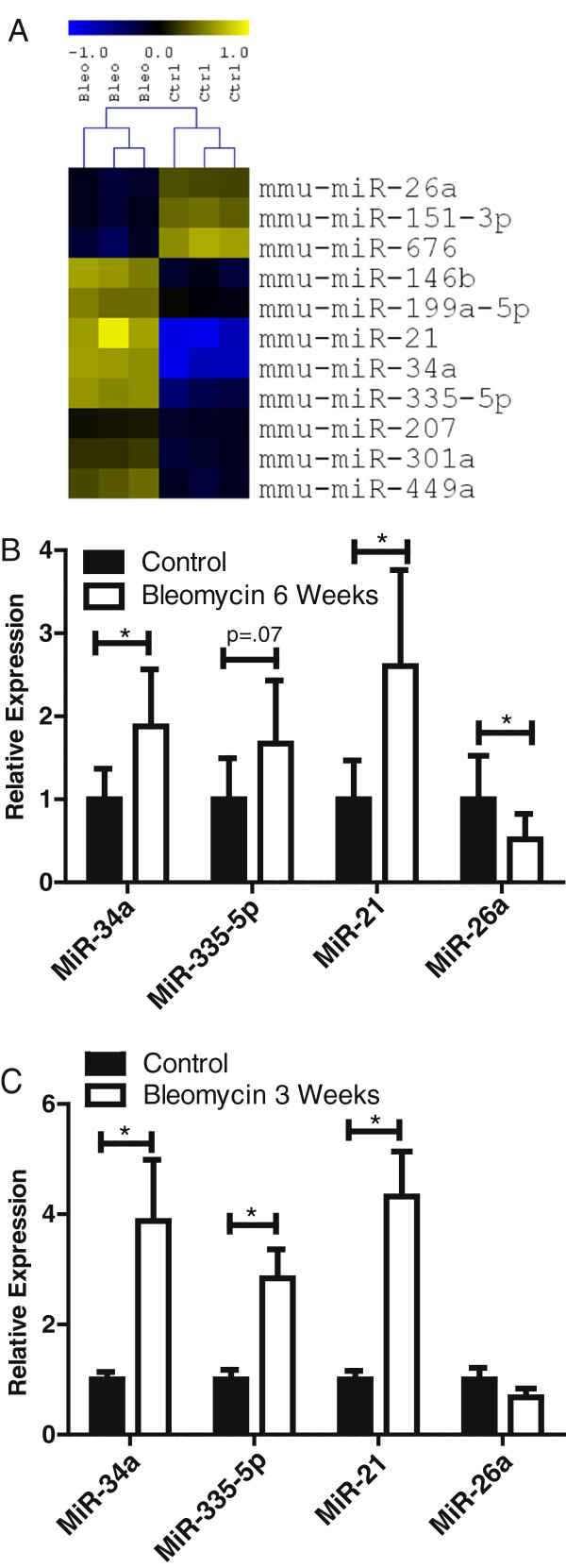
**Pulmonary microRNA profile of bleomycin treated and control C57BL/6J mice.** Mice were treated with 100 U/kg bleomycin through mini-osmotic pumps and lung tissue harvested three or six weeks later. **(A)** 11 microRNA were identified as being differentially expressed (FDR < 0.01) in lung clustering the treated and control mice separately. Relative expression is log_2_ transformed. Yellow indicates over expression, blue indicates under expression compared to a reference expression level. N = 3 mice per group. **(B)** MicroRNA expression in the lungs of bleomycin treated at six weeks and control mice, relative to the U6 control, was assessed by qRT-PCR. **(C)** MicroRNA expression in the lungs of bleomycin treated at three weeks and control mice, relative to U6 control, was assessed by qRT-PCR. Average ± standard deviation of n = 5 to 8 mice per group. * indicates a significant difference between groups, *P* < 0.05.

To determine whether the microRNA expression profile was altered in the fibrotic lung, we harvested lung tissue from mice six weeks after bleomycin treatment and from control mice and completed microarray analysis of microRNA expression levels. As shown in Figure 2, 11 microRNAs were differentially expressed (false discovery rate (FDR) < 0.01) between these groups, and the expression values of these microRNAs in lung tissue segregated the samples from fibrotic and control mice. Three microRNAs had decreased expression in the bleomycin treated lungs (miR-26a, miR-151-3p and miR-676) while eight microRNAs had increased expression in the bleomycin treated lungs (miR-146b, miR-199a-5p, miR-21, miR-34a, miR-335-5p, miR-207, miR-301a and miR-449a).

To verify the differentially expressed microRNA identified by microarray, we completed a quantitative RT-PCR assessment of miR-21, miR-34a, miR-355-5p, and miR-26a using lung RNA from biological replicates for mice six weeks after bleomycin treatment and for control mice, as shown in Figure 2B. When comparing bleomycin treated levels to control, miR-21 (*P* = 0.009), miR-34a (*P* = 0.015) and miR-355-5p (*P* = 0.07) were all increased, while miR-26a (*P* = 0.04) was decreased, confirming the microarray results. To determine whether these microRNAs were of altered expression earlier in the development of the phenotype, we completed quantitative RT-PCR on samples procured from mice at the three week timepoint. As seen in Figure 2C, miR-21, miR-34a and miR-355-5p were all significantly increased compared to controls, while miR-26a was not significantly decreased at this earlier timepoint.

**Figure 3:**
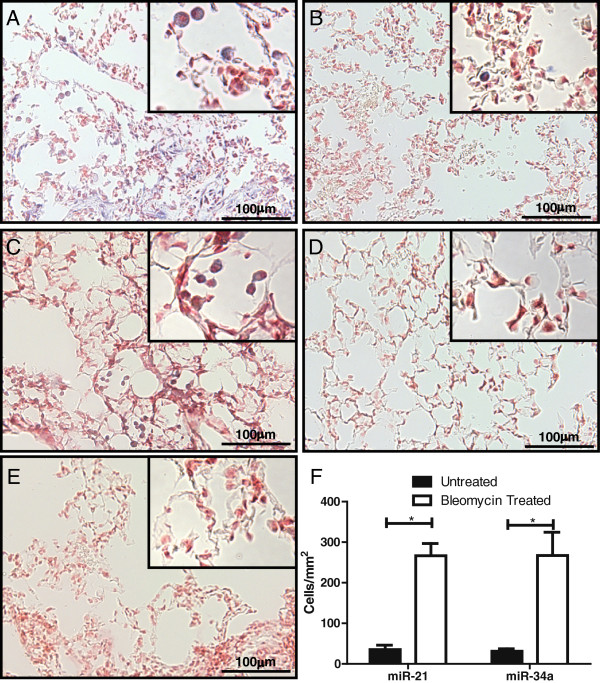
**Pulmonary expression of miR-21 and miR-34a in bleomycin treated and control C57BL/6J mice.** Mice were treated with100 U/kg bleomycin through mini-osmotic pumps and lung tissue harvested six weeks later. *In situ* hybridization of miR-21 in **(A)** bleomycin treated lungs and **(B)** control lungs. *In situ* hybridization of miR-34a in **(C)** bleomycin treated lungs and **(D)** control lungs. **(E)***In situ* hybridization using a scrambled probe as a negative control. No positive cells were identified when using the scrambled probe in control or bleomycin treated lungs. Magnification 400×, insert magnification 1000×. **(F)** Quantification of 10 random fields per lung for miR-21 and miR-34a positive cells per mm^2^ ± standard deviation of n = 4 to 9 mice per group. * indicates a significance difference between groups, *P* < 0.005.

*In situ* hybridization was conducted to histologically assess the levels of miR-21 and miR-34a in the lungs of mice at six weeks after bleomycin treatment and in control mice. As shown in Figure 3, the numbers of each of miR-21 and miR-34a positive cells were significantly increased in the lungs of bleomycin treated mice as compared to controls. The majority of miR-21 and miR-34a positive cells were within the alveolar space and were morphologically identified as macrophages, as seen in the magnified inserts. Further, immunohistochemical staining of the lungs of bleomycin treated and control animals showed an increase in F4/80 positive cells (macrophages) within the alveolar space at six weeks following bleomycin treatment, which corresponded to the increase in miR-21 and miR-34a positive cells (Additional file 1).

### Functional analysis of microRNA targets

To evaluate the potential biological consequence of the differentially expressed pattern of microRNAs in fibrotic lung tissue, we initially compiled a list of genes predicted to be regulated by the significantly differentially expressed microRNAs (n = 11; Figure 2) by TargetScan. In addition, we had previously measured the gene expression profile of bleomycin-induced pulmonary fibrosis in C57BL/6J mice also using mini-osmotic pumps, and evaluated at six weeks [33]. An assessment of the extent of overlap of these predicted targets with the measured signature of differentially expressed genes revealed 195 of the 2,527 predicted target genes to be common, and in the correct orientation, with genes which were differentially expressed in the lungs of bleomycin treated mice (n = 1,954). The overlap was considered to be in the correct orientation when a gene targeted by an upregulated microRNA was decreased in the gene expression profile, or when a gene targeted by a downregulated microRNA was increased in the gene expression profile.

Pathway analysis of the 195 genes that were predicted targets of the microRNAs and present in the gene expression analysis revealed that microRNAs potentially affect, among others, hepatocyte growth factor (HGF) signaling, insulin-like growth factor 1 (IGF-1) signaling and molecular mechanisms of cancer pathways in bleomycin-induced pulmonary fibrosis (Table 1). Genes that had altered expression but were not predicted to be influenced by microRNA levels were prominent in pathways including granulocyte adhesion and diapedesis, complement system and production of nitric oxide and reactive oxygen species in macrophages (Additional file 2).

**Table 1:** Pathways represented within the microRNA targets common to the set of bleomycin-induced genes

**Ingenuity canonical pathway**	**Minus log ( *P *-value)**	**Molecules**
Axonal guidance signaling	4.5	EPHA7, ARHGEF12,SOS2,PTCH1,ITGA5,ROBO1,WNT2,EFNB2,FZD4,IGF1,EFNB1,GNA13,PRKD3,ADAM9,NRP1
Role of NANOG in mammalian embryonic stem cell pluripotency	3.65	SOX2,LIF,FZD4,GAB1,SOS2,BMPR2,WNT2
HGF signaling	3.07	ETS1,GAB1,SOS2,MAPK10,ETS2,PRKD3
Cholecystokinin/gastrin-mediated signaling	3.02	ITPR2,SOS2,MAPK10,RHOU,GNA13,PRKD3
Pantothenate and CoA biosynthesis	2.93	PANK1,ENPP1,ENPP5
Molecular mechanisms of cancer	2.82	ARHGEF12,FZD4,GAB1,SOS2,PTCH1,MAPK10,RHOU,BMPR2,HIF1A,GNA13,PRKD3
Endothelin-1 signaling	2.56	EDNRB,GAB1,ITPR2,MAPK10,MAPK6,GNA13,PRKD3
RAR activation	2.49	TAF4 RARB,IGFBP3,MAPK10,NCOR1,PRKD3,PPARGC1A
Mouse embryonic stem Cell pluripotency	2.37	SOX2,LIF,FZD4,SOS2,BMPR2
Phospholipase C signaling	2.35	ARHGEF12,ITPR2,SOS2,RHOU,RPS6KA3,ITGA5,GNA13,PRKD3
IGF-1 signaling	2.31	CTGF,IGF1,SOS2,IGFBP3,GRB10
Hepatic fibrosis/hepatic stellate cell activation	2.31	COL1A2,CTGF,IGF1,EDNRB,FLT1,IGFBP3
ERK5 signaling	2.29	LIF,GAB1,RPS6KA3,GNA13
Glioblastoma multiforme signaling	2.17	FZD4,IGF1,ITPR2,SOS2,RHOU,WNT2
Growth hormone signaling	2.15	IGF1,IGFBP3,RPS6KA3,PRKD3
GDNF family ligand-receptor interactions	2.15	GAB1,ITPR2,SOS2,MAPK10
Renal cell carcinoma signaling	2.11	ETS1,GAB1,SOS2,HIF1A

**Figure 4:**
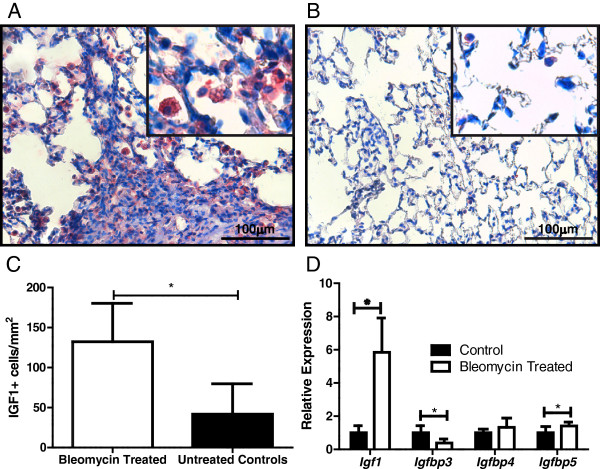
**Pulmonary expression of IGF-1 pathway genes in bleomycin treated and control C57BL/6J mice.** Immunohistochemistry of Igf1 in **(A)** bleomycin treated lungs and **(B)** control lungs. Magnification 400×, insert magnification 1000×. **(C)** Quantification of Igf-1 positive cells per mm^2^ lung tissue ± standard deviation of n = 4 mice per group. **(D)** qRT-PCR of lung tissue from bleomycin treated and control mice for genes of the IGF-1 pathway. Expression is relative to reference gene *Ataxin 10*. Average ± standard deviation of n = 6 to 8 mice per group. * indicates a significant difference between groups, *P* < 0.05.

To investigate whether the IGF-1 signaling pathway was altered in this model of bleomycin-induced lung disease, we assayed the expression of IGF family members in lung with qRT-PCR and immunohistochemistry. As shown in Figure 4, both *Igf-1* and *Igfbp5* were significantly increased in lung tissue from mice at six weeks after bleomycin treatment when compared to control, while *Igfbp3* was significantly decreased. Supporting this, the number of Igf-1 positive cells was significantly increased in pulmonary tissue from bleomycin treated mice. The Igf-1 positive cells were morphologically consistent with macrophages.

## Discussion

In this study, we provide evidence for a set of microRNAs which are of altered expression in pulmonary tissue of mice challenged with bleomycin by mini-osmotic pump, and we specifically show miR-21 and miR-34a to be predominately expressed in lung macrophages in this model of pulmonary fibrosis. Secondly, through bioinformatic analysis of the predicted targets and of genes known to have altered expression in bleomycin treated mice, pathways through which the microRNAs could affect lung disease were revealed. Among these we identified the IGF-1 pathway as putatively regulated by microRNAs in lung fibrosis and showed that numbers of Igf-1 positive cells, also macrophages, were increased in the lungs of bleomycin treated mice.

Through expression profiling, we identified 11 microRNAs to be differentially expressed in the lungs of mice presenting bleomycin-induced pulmonary fibrosis compared to lungs from untreated control mice and of these six have been previously reported in bleomycin response models. In detail, Liu *et al*. [29] profiled lung tissue from mice 7 and 21 days following exposure to intratracheal bleomycin and among the microRNAs of altered expression were increased levels of miR-21, miR-34a and decreased levels of miR-26a, in concordance with our data. Using a model of intraperitoneal delivery of bleomycin, Cushing *et al*. [30] reported the altered expression of additional microRNAs common to the present work, miR-449a and miR-146b, further to their evidence of miR-21, miR-34a within the fibrosis microRNA profile at 10 and 28 days following bleomycin administration. Finally, Lino Cardenas *et al*. [28] showed these four microRNAs, as well as miR-199a-5p to be among the microRNAs differentially expressed in the lungs of mice which developed fibrosis 14 days after intratracheal bleomycin instillation. Further work in each of these studies demonstrated specific microRNAs (mir-21, mir-29 and mir-199a-5p) to be expressed in myofibroblasts, and to affect TGF-β signaling and fibroblast function, leading to fibrosis development.

Our findings which indicate miR-21 and miR-34a to be predominantly expressed in macrophages, a significant inflammatory component of our model [32], and others [30] suggest that microRNA regulation of inflammation may be important in the pathology of pulmonary fibrosis. Supporting these data, Lu *et al*. [21] also detected miR-21 as being expressed in pulmonary macrophages of *A. fumigatus*-challenged mice and in a survey of expression, the levels of miR-21 in macrophages exceeded that of epithelial or fibroblast cell lines. Secondly, Vaporidi *et al*. [35] reported miR-21 to be expressed in macrophages in a mouse model of ventilator-induced lung injury.

The profile of differentially expressed microRNAs in this model of bleomycin-induced lung disease includes specific microRNAs which have been functionally implicated in mechanisms of relevance to fibrosis development. For example, miR-34a has been shown to regulate cardiac function and ageing, in part through affecting fibrosis in this tissue [36] and miR-26a can also affect cardiac fibrosis through altering collagen I levels [37]. MiR-146b has been shown to respond to TGF-β signaling [38], which is a key pro-fibrotic cytokine [8,39], in a model of intestinal epithelial cell differentiation. Finally, miR-301a was demonstrated to contribute to T helper type 17 cell development in a model of autoimmune encephalomyelitis [40], and thus could function to alter this lymphocyte of importance to pulmonary fibrosis pathology [41].

The analysis of the bleomycin-induced pulmonary fibrosis gene expression profile, combined with that of the induced changes in pulmonary microRNA levels, revealed novel pathways through which pulmonary fibrosis may develop in this model. In particular, microRNA regulation of genes of hepatocyte growth factor-, endothelin-1- or IGF1- signaling, and specific molecular mechanisms of cancer, may affect lung fibrosis. Using the combined microRNA-mRNA approach employed here, Dong *et al*. [42] implicated mir-29 mediated effects on the cell cycle and on cell adhesion, among other processes, to influence bronchopulmonary dysplasia in mice. Secondly, using this type of analysis, Ezzie *et a*l. [22] revealed that microRNA regulation of transforming growth factor β, Wnt and focal adhesion pathways may be relevant to the development of clinical chronic obstructive pulmonary disease.

IGF-1 signaling was among the pathways revealed in the gene expression analysis and there is both clinical [43,44] and experimental [45,46] evidence that perturbations of IGF signaling could contribute to IPF. Specifically, increased IGF-1 [43] and IGFBP5 [44] levels were reported for lung samples from IPF patients compared to controls and Uh *et al*. [47] demonstrated macrophages to be the important source of IGF in IPF, which is consistent with the findings of our model. IGFBP3, which has decreased mRNA expression in our model, has been shown to be increased in patients with IPF in both bronchoalveolar lavage [43] and lung tissue [44], indicating that some components of the IGF1 pathway differ between human IPF and our model. Experimentally, Andronegui *et al*. [45] have shown adenoviral treatment of mice with both Igf and Tgf-β to increase pulmonary fibrosis over mice receiving Tgf-β alone, while Yasuoka *et al*. [46] demonstrated adenoviral treatment with Igfbp5 to have the same effect, thus directly implicating the IGF-1 pathway in fibrosis development. Ruan and Ying [48] suggest that this change in IGF binding proteins could be an initiating factor in IPF and Veraldi and Feghali-Bostwick [49] propose IGF binding proteins to be central mediators of fibrosis. Our findings, indicting the IGF pathway to be significantly represented in microRNA regulation of bleomycin-induced pulmonary fibrosis, coupled with increased Igf-1 levels in fibrotic lung tissue, support this line of investigation and suggest the involvement of microRNA regulation.

As by definition, the cause of idiopathic pulmonary fibrosis (IPF) is unknown, developing an animal model which accurately represents the disease has proven to be difficult [50]. Several methods exist to induce pulmonary fibrosis in rodents including modulation of gene expression using viral vectors or transgenic animals or administration of agents such as bleomycin, fluorescein isothiocyanate (FITC), silica, and irradiation [50,51]. Each of these models has strengths, but the majority fail to reproduce the chronic nature of IPF [50]. Despite limitations, bleomycin-induced fibrosis remains the most widely used and is considered to be the best model for the study of IPF [51,52]. There are many routes of bleomycin administration used in animal models including intratracheal, intravenous, intraperitoneal, or subcutaneous and each of these produces fibrosis at a different time point following treatment. While single doses of bleomycin are often sufficient to induce fibrosis, models that involve repeated or prolonged bleomycin exposure, such as our mini-osmotic pump, are considered improved [50,51] as they result in progressive fibrosis which more closely mimics IPF [3,32]. A limitation associated with this approach is the fact that the presence of the pump itself may affect the lung, and although we have shown that saline filled pumps do not produce pulmonary fibrosis in mice [53] we can not exclude an effect of the pumps on microRNA expression in this model.

## Conclusions

In conclusion, using microRNA profiling of a mini-osmotic pump model of bleomycin-induced pulmonary fibrosis, combined with gene expression profiling data, we have identified that microRNAs putatively affect the IGF-1 pathway in pulmonary fibrosis. Further, the finding of miR-21 and miR-34a expression in macrophages suggests microRNA regulation of the inflammatory response may contribute to the development of pulmonary fibrosis in this model.

## Methods

### Mice, bleomycin treatment and fibrosis phenotyping

C57BL/6J mice were purchased from the Jackson Laboratory (Bar Harbor, ME, USA) and housed at the Meakins-Christie Laboratories. At eight weeks of age, the mice were treated with 100 Units/kg bleomycin sulphate (Mayne Parma, Montreal, QC, Canada) dissolved in saline, through mini-osmotic pumps (Alzet 2001, Cupertina, CA, USA) as in past studies [33,34]. Untreated mice were assessed as controls. At three or six weeks post treatment, the mice were euthanized by sodium pentobarbital overdose. The right lung was immediately homogenized in TRI Reagent (Sigma, Oakville, ON, Canada) and stored at −80°C until RNA isolation [54,55]. The left lung of each mouse was perfused with 10% neutral buffered formalin and submitted for histological processing. Lung sections were stained with Masson’s trichrome to identify the area of collagen deposition in the lung which were determined from user-drawn regions and compared to the area of the entire lobe (Image-Pro Plus Software, Rockville, MD, USA) to generate the per cent fibrosis in the lung as in previous studies [53]. Animal experiments were completed under a protocol approved by the McGill University Animal Care Committee in agreement with the guidelines of the Canadian Council on Animal Care.

### RNA isolation and microarray

Total RNA from the lungs of three bleomycin treated animals and three untreated animals was isolated using miRNeasy Mini kits according to the manufacturer’s protocol (Qiagen, Germantown, MD, USA). Exiqon (Vedbaek, Denmark) performed target preparation and array hybridization according to their protocol. In short, 1 μg of total RNA from sample and reference was fluorescently labeled with Hy3 or Hy5 respectively and hybridized to a miRCURY LNA array version 11.0 (Exiqon, Vedbaek, Denmark) containing probes for all mouse microRNAs registered in miRBASE (version 12) [56]. 598 microRNA probes were assessed in quadruplicate with hybridization being performed on a Tecan HS480 hybridization station (Tecan, Männedorf, Switzerland). Slides were scanned using an Agilent G2565BA Microarray Scanner System (Agilent Technologies, Inc., Mississagauga, ON, Canada) and image analysis was carried out using ImaGene 8.0 software (BioDiscovery, Inc., Hawthorne, CA, USA). Data were background corrected and normalized using the global Lowess (LOcally WEighted Scatterplot Smoothing) regression algorithm [57]. Differential microRNA expression between bleomycin treated and control tissue was determined by Student’s two-tailed *t*-tests with *P* < 0.01 after FDR (false discovery rate) correction for multiple testing. The dataset was deposited into Genome Expression Omnibus (GEO; accession number GSE45789).

### Gene expression profile

Using previously published microarray data (GDS1492, [33]), the differential gene expression profile between bleomycin treated and control lung tissue was determined by Cyber-T test [58] with *P* < 0.01 after FDR correction for multiple testing.

### MicroRNA target prediction and pathway analysis

Predicted targets for the 11 significantly differentially expressed microRNAs were identified using TargetScan Human 6.0 [59]. This database predicts mouse genes using orthologs to human annotation owing to the improved documentation of the 3′-untranslated region of human genes. The gene expression profile of bleomycin-induced pulmonary fibrosis was previously published (GDS1492, [33]) and we filtered the genes from this list to determine gene expression that had an inverse relationship with microRNA expression levels.

Pathway analysis was completed by uploading gene lists into the Ingenuity Pathway Analysis program (Ingenuity® Systems, Redwood, CA, USA) and identifying the significant pathways represented in this list by application of Fisher’s exact test which calculates a *P*-value determining the probability that the association between the genes in the list and the database pathway were explained by chance alone. The significance threshold of pathways was set to 2 (derived by –log10 (P value), for *P* = 0.01).

### Quantitative real time PCR

Expression levels of specific microRNAs were analyzed by quantitative real time PCR (qRT-PCR) using miRCURY LNA™ microRNA PCR system according to the manufacturer’s protocol (Exiqon, Vedbaek, Denmark). RNA was converted to cDNA using a universal cDNA synthesis kit (Exiqon, Vedbaek, Denmark). cDNA samples were then PCR amplified using SYBR Green master mix and LNA™ microRNA primers (Exiqon, Vedbaek, Denmark). Primers targeting mmu-miR-335-5p (target sequence UCAAGAGCAAUAACGAAAAAUGU), mmu-miR-34a (target sequence UGGCAGUGUCUUAGCUGGUUGU), mmu-miR-21 (target sequence UAGCUUAUCAGACUGAUGUUGA) and mmu-miR-26a (target sequence UUCAAGUAAUCCAGGAUAGGCU) were used. Samples were run on an Applied Biosystems International Prism 7500 instrument (Burlington, ON, Canada). The data were normalized to a U6 RNA control and relative expression was calculated using the comparative C_T_ method, as previously described [60].

Gene expression experiments were completed as described previously [61]. Briefly, 4 to 5 μg of total RNA from the right mouse lung was reversely transcribed with oligo(dT) primer using Superscript™ III RNase H-Reverse Transcriptase (Invitrogen, Carlsbad, CA, USA) to make cDNA. Quantitative real-time PCR assays were performed using the Applied Biosystems International Prism 7500 Sequence Detection System and assays on demand for *Igf1* (Insulin-like growth factor 1, Mn00439561_m1), *Igfbp3* (Insulin-like growth factor-binding protein 3, Mn01187817_m1), *Igfbp4* (Mn00516037_m1) and *Igfbp5* (Mn00494922_m1), with *Ataxin 10* (Atxn10, assay Mm00450332_m1) used as the reference gene. Relative expression was calculated using the comparative C_T_ method [60] and differences between bleomycin treated and control animals were assessed by Student’s *t*-tests.

### *In-situ* hybridization

*In situ* hybridization was performed using miRCURY LNA™ ISH Optimization Kit (Exiqon, Vedbaek, Denmark) according to the manufacturer’s instructions. Briefly, lung sections were incubated with Proteinase K to expose microRNAs, and hybridized with double-digoxigenin labeled probes against miR-21 and miR-34a (Exiqon, Vedbaek, Denmark). Digoxigenin-labeled probes were detected with sheep anti-digoxigenin-alkaline phosphatase antibody (Roche, Mississauga, ON, Cnada) and visualized with nitro blue tetrazolium chloride/5-bromo-4-chloro-3-indolyl phosphate ready-to-use tablets (Roche, Mississauga, ON, Canada). Slides were counterstained with Nuclear Fast Red (Sigma, Oakville, ON, Canada). Scoring of 10 complete fields randomly selected throughout each lung was performed by a user blind to the treatment and presented as the average number of positively stained cell/mm^2^. Differences between groups were assessed with Student’s *t*-test.

### Immunohistochemistry

Staining was completed as previously described [62,63] using antibodies against Igf1 (Santa Cruz Biotechnology Inc., Santa Cruz, CA, USA, sc-9013, dilution 1:50) and F4/80 (Serotec, Raleigh, NC, USA, MCA497R, dilution 1:300). Sections were developed using Vectastain ABC-Alkaline phosphatase kit (Vector Laboratories, Burlington, ON, Canada) and Vector Red Alkaline phosphatase substrate kit (Vector Laboratories, Burlington, ON, Canada). Slides were counterstained with methyl green. Blind scoring of 10 complete fields randomly selected throughout each lung was performed and presented as the average number of positively stained cell/mm^2^. Differences between groups were assessed with Student’s *t*-test.

## Abbreviations

FDR: False discovery rate; FITC: Fluorescein isothiocyanate; HGF: Hepatocyte growth factor; IGF-1: Insulin-like growth factor; Igfbp3: Insulin-like growth factor-binding protein 3; Igfbp4: Insulin-like growth factor-binding protein 4; Igfbp5: Insulin-like growth factor-binding protein 5; IPF: Idiopathic pulmonary fibrosis; miR: microRNA; qRT-PCR: Quantitative real time polymerase chain reaction; TGF- β1: Transforming growth factor β1.

## Competing interests

The authors declare that they have no competing interests.

## Authors’ contributions

LH carried out the histological studies, data analysis and manuscript preparation. MB performed qRT-PCR, pathway analysis, data analysis and manuscript preparation. TGT contributed to the pathway analysis and data analysis. CKH conceived of the study, participated in the design and drafted the manuscript. All authors read and approved of the final manuscript.

## Supplementary Material

Additional file 1 **F4/80 positive cells in bleomycin treated and control C57BL/6J mice.** Immunohistochemistry of F4/80 in **(A)** bleomycin treated lungs and **(B)** control lungs. Magnification 400×, insert magnification 1000×. **(C)** Quantification of F4/80 positive cells per mm^2^ lung tissue ± standard deviation of n=7-13 mice per group. * indicates a significant difference between groups, p < 0.0005. Click here for file

Additional file 2 Pathways significantly represented within differentially expressed genes that were not predicted to be regulated by microRNA. Click here for file
