# Supplementary figures and images for: MicroRNA profiling implicates the insulin-like growth factor pathway in bleomycin-induced pulmonary fibrosis in mice
Source: Fibrogenesis Tissue Repair. 2013 Aug 29;6:16. doi: 10.1186/1755-1536-6-16 (PMC3766165; doi:10.1186/1755-1536-6-16)

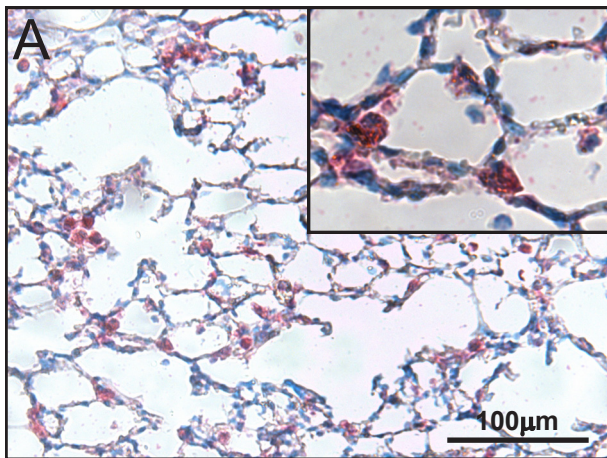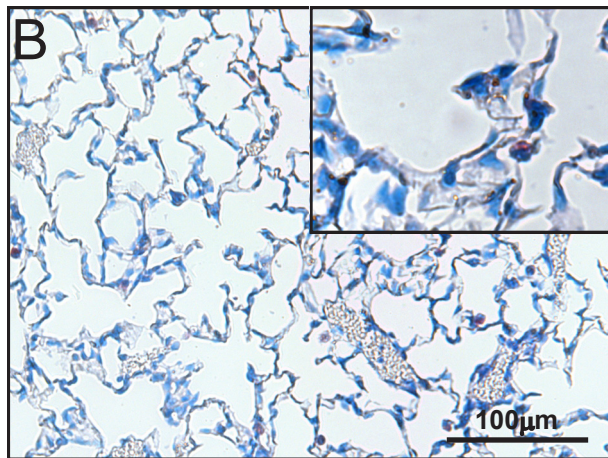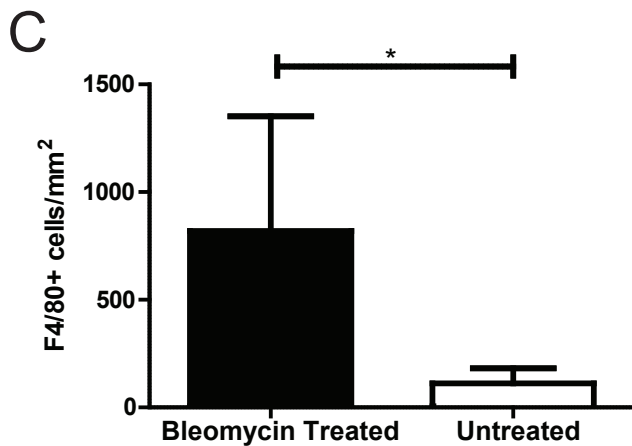

Supplement: Additional file 1 — F4/80 positive cells in bleomycin treated and control C57BL/6J mice. Immunohistochemistry of F4/80 in (A) bleomycin treated lungs and (B) control lungs. Magnification 400×, insert magnification 1000×. (C) Quantification of F4/80 positive cells per mm2 lung tissue ± standard deviation of n=7-13 mice per group. * indicates a significant difference between groups, p < 0.0005. [file 1755-1536-6-16-S1.pdf]
