# Supplementary material for: MicroRNA profiling implicates the insulin-like growth factor pathway in bleomycin-induced pulmonary fibrosis in mice
Source: Fibrogenesis Tissue Repair. 2013 Aug 29;6:16. doi: 10.1186/1755-1536-6-16 (PMC3766165; doi:10.1186/1755-1536-6-16)
Supplement: Additional file 2 — Pathways significantly represented within differentially expressed genes that were not predicted to be regulated by microRNA. [file 1755-1536-6-16-S2.pdf]

Supplementary Table 1: Pathways significantly represented within differentially expressed genes that were not predicted to be regulated by microRNA.

Ingenuity Canonical Pathway	-log (p-value)
Agranulocyte Adhesion and Diapedesis	13.80
Granulocyte Adhesion and Diapedesis	13.60
Hepatic Fibrosis / Hepatic Stellate Cell Activation	10.10
Atherosclerosis Signaling	7.40
Leukocyte Extravasation Signaling	5.12
Inhibition of Matrix Metalloproteases	4.80
LXR/RXR Activation	4.60
Production of Nitric Oxide and Reactive Oxygen Species in Macrophages	4.26
Nicotine Degradation II	4.09
Complement System	4.03
Inhibition of Angiogenesis by TSP1	3.91
Glutathione-mediated Detoxification	3.90
RhoA Signaling	3.88
Histamine Degradation	3.74
Ethanol Degradation IV	3.73
IL-8 Signaling	3.66
ILK Signaling	3.62
LPS/IL-1 Mediated Inhibition of RXR Function	3.41
IL-10 Signaling	3.40
Eicosanoid Signaling	3.35
Role of Pattern Recognition Receptors in Recognition of Bacteria and Viruses	3.19
Oxidative Ethanol Degradation III	3.16
Intrinsic Prothrombin Activation Pathway	3.14
Aryl Hydrocarbon Receptor Signaling	2.85
Colorectal Cancer Metastasis Signaling	2.80
Bladder Cancer Signaling	2.71
Bupropion Degradation	2.64
Ethanol Degradation II	2.63
Dendritic Cell Maturation	2.56
Prostanoid Biosynthesis	2.55
Acetone Degradation I (to Methylglyoxal)	2.54
Nicotine Degradation III	2.49
p53 Signaling	2.47
Actin Cytoskeleton Signaling	2.35
Glycolysis I	2.25
Putrescine Degradation III	2.19
Acute Phase Response Signaling	2.18
4-1BB Signaling in T Lymphocytes	2.18
Arginine Degradation VI (Arginase 2 Pathway)	2.16
Dopamine Degradation	2.16
NRF2-mediated Oxidative Stress Response	2.13
Cardiac β-adrenergic Signaling	2.05
Guanosine Nucleotides Degradation III	2.03
